# The bioactivity of plant extracts against representative bacterial pathogens of the lower respiratory tract

**DOI:** 10.1186/1756-0500-2-95

**Published:** 2009-06-01

**Authors:** Virgilio Bocanegra-García, María del Rayo Camacho-Corona, Mónica Ramírez-Cabrera, Gildardo Rivera, Elvira Garza-González

**Affiliations:** 1 Departamento de Biología Molecular y Bioingeniería, UAM Reynosa Aztlán, Universidad Autónoma de Tamaulipas, Reynosa, Mexico; 2 Laboratorio de Química de Productos Naturales, División de Estudios Superiores de la Facultad de Ciencias Químicas, Universidad Autónoma de Nuevo León, Monterrey, NL, Mexico; 3 Departamento de Microbiología Facultad de Medicina, Universidad Autónoma de Nuevo León, Monterrey, NL, Mexico

## Abstract

**Background:**

Lower respiratory tract infections are a major cause of illness and death. Such infections are common in intensive care units (ICU) and their lethality persists despite advances in diagnosis, treatment and prevention. In Mexico, some plants are used in traditional medicine to treat respiratory diseases or ailments such as cough, bronchitis, tuberculosis and other infections. Medical knowledge derived from traditional societies has motivated searches for new bioactive molecules derived from plants that show potent activity against bacterial pathogens. Therefore, the aim of this study was to evaluate the effect of hexanic, chloroformic (CLO), methanolic (MET) and aqueous extracts from various plants used in Mexican traditional medicine on various microorganisms associated with respiratory disease.

**Methods:**

thirty-five extracts prepared from nine plants used in Mexican traditional medicine for the treatment of respiratory infections were evaluated against 15 control bacterial species and clinical isolates.

**Results:**

Both chloroformic (CLO) and methanolic (MET) extracts of *Larrea tridentata *were active against Methicillin-resistant *S. aureus*, *B. subtilis *and *L. monocytogenes*. A MET extract of *L. tridentata *was also active against *S. aureus*, *S. pneumoniae*, *S. maltophilia*, *E. faecalis *and *H. influenzae *and the CLO extract was active against *A. baumannii*. An Aqueous extract of *M. acumitata *and a MET extract of *N. officinale *were active against *S. pneumoniae*. CLO and MET extracts of *L. tridentata *were active against clinical isolates of *S. aureus*, *S. pneumoniae *and *E. faecalis*.

**Conclusion:**

Overall, our results support the potential use of *L. tridentata *as a source of antibacterial compounds.

## Findings

Lower respiratory tract infections are a major cause of illness and death and are common in intensive care units (ICU) [1,2]. The most frequent causal agents of these infections are *Streptococcus pneumoniae, Klebsiella pneumoniae, Staphylococcus aureus, Haemophilus influenzae, Pseudomonas aeruginosa, Acinetobacter baumannii *and *Stenotrophomonas maltophilia*. Some additional and less frequent causative agents are *Escherichia coli*, *Enterobacter cloacae*, *Bacillus subtilis *[3], *Bacillus cereus *[4], *Listeria monocytogenes *[5], and *Enterococcus faecalis *[6].

Infections with these bacteria are associated with high morbidity and mortality, especially in immunocompromised patients [7-10]. Methicillin-resistant *S. aureus *(MRSA) is of particular concern in the United States since approximately 60% of all staphylococcal infections in the ICU are caused by MRSA [11,12]. Penicillin-resistant *S. pneumoniae *and vancomycin-resistant enterococci are an additional cause for concern in the clinical setting [13].

In Mexico, some plants are used in traditional medicine to treat respiratory diseases or ailments such as cough, bronchitis, tuberculosis and other infections [14,15]. The known success of these traditional therapies has guided the search for new chemotherapeutic alternatives to fight respiratory and other infections caused by drug-resistant bacteria [16-18]. Therefore, the aim of this study was to evaluate the effect of hexanic, chloroformic (CLO), methanolic (MET) and aqueous extracts from various plants used in Mexican traditional medicine on various microorganisms associated with respiratory disease.

### Plant material and preparation of extracts

We reviewed Mexican ethnobotanical literature and selected a group of plants used by traditional healers to treat respiratory infections [14,15]. In 2004, nine plants were collected by the biologist Mauricio Gonzalez-Ferrara from different states of Mexico, and authenticated by the Biologist Marcela González-Alvarez. Voucher specimens were deposited at the UANL herbarium located in the Facultad de Ciencias Biológicas of the Universidad Autonoma de Nuevo Leon, Mexico.

Plant names, author of the name, plant family, parts used, voucher number, and the state where collected were as follows: *Citrus aurantifolia *(Christm) Swingle (Rutaceae), fruit peel, 024769, Nuevo Leon; *Citrus sinensis *(L) Osbeck (Rutaceae), fruit peel, 024770, Nuevo Leon; *Foeniculum vulgare *P. Mill (Umbelliferae), aerial parts, 024771, Chihuahua; *Larrea tridentata *(Sessé & Moc. ex. DC) Coville (Zygophyllaceae), aerial parts, 024772, Nuevo Leon; *Nasturtium officinale *R. Br. (Cruciferae), aerial parts, 024774, Nuevo Leon; *Olea europaea *L (Oleaceae), leaves, 024775, Nuevo Leon; *Mentha pulegium *L. (Labiatae), leaves, 024777, purchased in the Sonora Market in Mexico City; *Rosa centifolia *L. (Rosaceae), petals, 024776, Morelos and *Musa acuminata *Colla (Musaceae), stems, 024773, Guerrero.

Approximately 50 g of dried and ground plant material was successively extracted by maceration with hexane, chloroform, methanol and water. Organic extracts were concentrated *in vacuo *to dryness, and aqueous extracts were freeze-dried. All extracts were stored at 4°C until use.

Hexanic, CLO, MET and aqueous extracts were made from the following: *C. aurantifolia*, *C. sinensis*, *F. vulgare*, *L. tridentata*, *N. officinale*, *O. europaea*, *M. pulegium*, *R. centifolia*. Only CLO, MET and aqueous extracts were prepared form *M. acuminata *because of limited plant material availability.

Stock solutions were prepared from extracts at a concentration of 20 mg/ml. For organic extracts, DMSO was used as a solvent. Aqueous extracts were prepared in distilled water and sterilized by filtration through a 0.22 μm membrane.

### Bacterial strains and preparation of inocula

Two sets of control species or phenotypic groups of bacteria were tested. The Gram-negative bacteria were *S. maltophilia *ATCC 12714, *E. coli *ATCC 25922, *A. baumannii *ATCC 15308, *H. influenzae *ATCC 49766, *H. influenzae *ATCC 49247, *P. aeruginosa *ATCC 27853, *K. pneumoniae *ATCC 700603 and *E. cloacae *ATCC 35030. The Gram-positive strains were: *S. aureus *ATCC 29213, *S. aureus *MRSA ATCC BAA-44, *B. subtilis *ATCC 6633, *B. cereus *ATCC 49064, *S. pneumoniae *ATCC 49619, *L. monocytogenes *ATCC 19111 and *E. faecalis *ATCC 29212.

Before testing, strains were inoculated onto plates prepared with 5% blood agar and cultured overnight at 37°C. For *H. influenzae*, strains were inoculated onto chocolate agar and incubated in a 5% CO_2 _atmosphere for 48 h. Turbidity was adjusted to 0.5 of the McFarland standard to prepare the test inoculum. Afterwards, 10 μl were transferred into 11 ml Mueller Hinton broth to achieve 5 × 10^5 ^CFU/ml. HTM medium was used for *H. influenzae *and Mueller Hinton with TES/lysed horse blood was used for *S. pneumoniae*.

### Screening of antimicrobial activity and reference drugs

Minimal inhibitory concentration (MIC) for each extract was determined for both Gram-negative and Gram-positive bacteria by the microdilution method [19] with dilutions ranging from 250 to 7.8 μg/ml. Extracts that caused complete inhibition of growth at 24 h were considered active and used for the subsequent assays. All assays were performed in duplicate.

Two extracts with the broadest spectrum of activity were further tested against clinical isolates of susceptible species collected at the Hospital Universitario "Dr. José Eleuterio" Gonzalez and deposited at the Microbiology Department, School of Medicine, UANL. These isolates included *A. baumannii *(n = 25), *E. faecalis *(n = 15), *S. aureus *(n = 25), and *S. pneumoniae *(n = 13; total n = 78). *H. influenzae *was not included because clinical isolates were not available.

For all assays, adequate media without drug or extract was used as control.

As a reference for the screening assays, MICs for the antimicrobial drugs ceftazidime (Caz), ciprofloxacin, levofloxacin (Lv) and vancomycin (Va) were determined by the broth microdilution method recommended by the Clinical and Laboratory Standards Institute [20]. As a reference for the assays with clinical isolates, we evaluated the susceptibility pattern for each clinical isolate against the following commonly used antibiotics: Caz, Lv, Va, minocycline, penicillin, piperacillin tazobactam, amoxicillin clavulanic acid, ampicillin, linezolid, meropenem, amikacin, cefepime, and/or ceftriaxone.

## Results

CLO and MET extracts of *L. tridentata*, MET extract of *N. officinale *and aqueous extract of *M. acuminata *were all active against several Gram-positive bacteria and Gram-negative bacteria [see Additional file 1]. None of the 35 extracts were active against *B. cereus*, *E. coli*, *P. aeruginosa*, *K. pneumoniae *or *E. cloacae *strains at the tested concentrations. Hexanic, CLO, MET and aqueous extracts of *C. aurantiifolia*, *C. sinensis*, *F. vulgare*, *M. pulegium*, *O. europaea *and *R. centifolia *did not show activity against any of the 15 bacteria of phenotypic groups.

We selected the CLO and MET extracts of *L. tridentata *because of their broad bioactivity and evaluated their effects on clinical isolates of species that showed sensitivity to these extracts. The clinical isolates included were *A. baumannii *(n = 25), *E. faecalis *(n = 15), *S. pneumoniae *(n = 13) and *S. aureus *(n = 25). The most potent antibacterial activity occurred against the *S. pneumoniae *strains [see Additional file 2] with MIC_50 _and MIC_80 _values of 31.25 μg/ml for both CLO and MET extracts, respectively. These extracts also showed potent activity against *E. faecalis *(n = 15, MIC_50 _= 250 μg/ml for both extracts) and *S. aureus *(n = 25, MIC_80 _= 125 μg/ml for both extracts) [see Additional file 2]. No activity was detected against any clinical isolates of *A. baumannii *(n = 25).

## Discussion and conclusion

Among the plant extracts that showed antibacterial activity, the CLO and MET extracts of *L. tridentata *showed the broadest and most promising spectrum of activity. In order to confirm this activity, we tested those extracts against clinical isolates of species that showed sensitivity to these extracts in the screening assay.

Both extracts confirmed the antibacterial activity, not only in control strains, but also against strains isolated from patient's clinical samples. The best activity was observed against *S. pneumoniae *(MICs_80 _= 31.25 μg/ml). When tested against *S. pneumoniae*, the antimicrobial activity of CLO and MET extracts of *L. tridentata *were comparable to the activity observed for reference antimicrobial drugs. It is possible that further isolation of the active compound(s) will render lower MIC values.

Both the CLO and MET extracts of *L. tridentata *also showed good activity against *E. faecalis *(MICs_50 _= 250 μg/ml) and *S. aureus *(MICs_80_= 125 μg/ml). These results are quite interesting, given the high drug-resistance observed for those clinical isolates. The CLO and MET extracts of *L. tridentata *were active against MDR clones of *S. aureus*. These results are particularly important because of the increase in morbidity and mortality related to clones of MDR *S. aureus *in the last two decades [11].

No activity was detected against any clinical isolates of *A. baumannii*. Nevertheless, this is not a surprising result sinceclinical isolates of *A. baumannii *show a strong drug-resistance, so there are likely multiple antimicrobial-resistance mechanisms in these strains.

The CLO and MET extracts of *L. tridentata *showed different activity spectra. Verastegui et al. reported that ethanolic extract of *L. tridentata *was active against *L. monocytogenes, Clostridium perfringens, Shigella dysenteriae, Yersinia enterocolitica *and *Proteus vulgaris*, with a MIC ranging from 10 to 19 μg/ml, yet they did not find activity against *E. coli *[21]. We found that CLO and MET extracts of *L. tridentata *showed activity against *L. monocytogenes *and no activity against *E. coli*. This confirms the antibiotic activity of *L. tridentata *extracts; however, it is difficult to compare our results directly with Verastegui et al. because we did not include these ethanolic extracts in our study.

*L. tridentata *(Creosote bush) is an abundant plant of Mexican and US-American deserts. The potent antioxidant nordihydroguaiaretic acid (NDGA) has been identified from this plant [22]. This lignan is a potential therapeutic molecule for Sjorgren-Larsson syndrome, cardiopathies, skin cancer prevention, and viral infections [23]. We presume that the compounds present in our *L. tridentata *CLO extract are not NDGA because they have a different polarity. Thus, they are potentially novel antibacterial molecules. Antimicrobial properties for some plant species tested in the study have previously been described but not with a special focus on respiratory tract pathogens and the inclusion of some bacterial MDR clones.

Our results showed that the MET extract of *N. officinale *and the aqueous extract of *M. acuminata *were active against *S. pneumoniae. N. officinale *has been used for the treatment of renal, liver disease [24], and has also been shown to reduce oxidative stress in hypercholesterolemic rats [25]. This plant has also been shown to have cardio-protective activity [26] and antimycobacterial potential [27]. Indeed, several antimicrobial compounds have been identified in *N. officinale*. Specifically, a phenyl-phenalenone has been isolated and evaluated for leishmanicidal activity and isolated phytoalexins are known to have antifungal activity [28]. We found that the methanolic extract of *N. officinale *is active against *S. pneumoniae*.

Our results also demonstrated the activity of *M. acuminata *aqueous extract against *S. pneumoniae *ATCC 49619 (250 μg/ml). Similarly, activity of *M. acuminata *MET extract against *Mycobacterium tuberculosis *has been previously reported [27]. To our knowledge, there is no other antimicrobial activity report for extracts form *M acuminata*.

Our findings do conflict with some of the previously published results on the same plant extracts. We did not find antibacterial activity in several plants whose antimicrobial effects have been documented. For example, we did not find any antibacterial activity in extract from *O. europaea*, yet another group isolated the aldehyde hexanal extract from *O. europaea *and reported bioactivity against multiple types of microorganisms [29]. In addition, a terpenoid extracted from *C. sinensis *with antifungal activity has been isolated from the fruit peel [30], yet we found no effect of the extract in our study.

The differences between our study and theirs could have multiple explanations. The strains or clones and the testing concentrations differed and perhaps local environmental factors that affect the potency of medicinal plants, such as temperature, rainfall, day length and soil characteristics may have differed between the plant samples used for each study. Also, it is important to consider differences in the conditions of extract preparation and in the extract concentrations tested.

Our study has provided and confirmed previous evidence that *L. tridentata *is a promising alternative source for antimicrobial compounds. It is particularly valuable in the context of the bacterial resistance that is prevalent. Our group is currently conducting a study to isolate the active compounds in this extract.

Lastly, the activity observed for *L. tridentata *provides a rationale for its use in the treatment of respiratory infectious diseases in traditional medicine.

## List of abbreviations

CLO: Chlroformic; MET: Methanolic; ICU: Intensive Care Units; MDR: Multidrug-Resistant; MTC: Minimal inhibitory concentration.

## Competing interests

There authors declare that they have no competing interests.

## Authors' contributions

VBG, characterized the plant extracts with regard to the inhibitory effects on the selected strains, contributed with the study design and helped to draft the manuscript. MRCC, collected the plant material, and contributed with the study design. MARC, obtained the plant extracts. GRS, contributed with the study design and helped with the draft of the manuscript. EGG, designed the study, coordinated the experiments, and wrote the paper.

All authors read and approved the final manuscript.

## Supplementary Material

Additional file 1 **Table 1.** MIC values (μg/ml) of plant extracts relative to strains that were sensitive to at least one extract. Click here for file

Additional file 2 **Table 2.** Range, MIC_50_, MIC_80 _and MIC_90 _values (μg/ml) of *L. tridentata *extracts relative to clinical isolates and reference drug MIC_90 _values. Click here for file
