# Supplementary material for: The bioactivity of plant extracts against representative bacterial pathogens of the lower respiratory tract
Source: BMC Res Notes. 2009 Jun 1;2:95. doi: 10.1186/1756-0500-2-95 (PMC2702266; doi:10.1186/1756-0500-2-95)
Supplement: Additional file 1 — Table 1. MIC values (μg/ml) of plant extracts relative to strains that were sensitive to at least one extract. [file 1756-0500-2-95-S1.doc]

Table 1. MIC values (μg/ml) of plant extracts relative to strains that were sensitive to at least one extract.

| Plant/Family/Extract  Location/Plant part | *S. aureus* ATCC 29213 | *S. aureus* ATCC BAA-44 | *B. subtilis* ATCC 6633 | *S. pneumoniae* ATCC 49619 | *L. monocytogenes* ATCC 19111 | *E. faecalis* ATCC 29212 | *A. baumannii* ATCC 15308 | *S. maltophilia* ATCC 12714 | *H. influenzae* ATCC 49766 | *H. influenzae* ATCC 49247 |
| --- | --- | --- | --- | --- | --- | --- | --- | --- | --- | --- |
| *Larrea tridentata* (Zygophyllaceae) / Nuevo Leon / aerial parts | | | | | | | | | | |
| Hexanic | >250 | >250 | >250 | >250 | >250 | >250 | >250 | >250 | >250 | >250 |
| Chloroformic | >250 | 250 | 62.25 | >250 | 125 | >250 | 125 | >250 | >250 | >250 |
| Methanolic | 250 | 250 | 125 | 250 | 125 | 125 | >250 | 250 | 125 | 125 |
| Aqueous | >250 | >250 | >250 | >250 | >250 | >250 | >250 | >250 | >250 | >250 |
| *Musa acuminata* (Musaceae) / Guerrero / Steam | | | | | | | | | | |
| Chloroformic | >250 | >250 | >250 | >250 | >250 | >250 | >250 | >250 | >250 | >250 |
| Methanolic | >250 | >250 | >250 | >250 | >250 | >250 | >250 | >250 | >250 | >250 |
| Aqueous | >250 | >250 | >250 | 250 | >250 | >250 | >250 | >250 | >250 | >250 |
| *Nasturtium officinale* (Cruciferae) / Nuevo Leon / aerial parts | | | | | | | | | | |
| Hexanic | >250 | >250 | >250 | >250 | >250 | >250 | >250 | >250 | >250 | >250 |
| Chloroformic | >250 | >250 | >250 | >250 | >250 | >250 | >250 | >250 | >250 | >250 |
| Methanolic | >250 | >250 | >250 | 250 | >250 | >250 | >250 | >250 | >250 | >250 |
| Aqueous | >250 | >250 | >250 | >250 | >250 | >250 | >250 | >250 | >250 | >250 |
| Referente drugs | | | | | | | | | | |
| Levofloxacin |  |  | 2 | 4 | 2 | 2 | 4 | 4 | 1 | 1 |
| Vancomycin | 2 | 4 |  | 16 | 16 |  |  |  |  |  |
