# Supplementary material for: The bioactivity of plant extracts against representative bacterial pathogens of the lower respiratory tract
Source: BMC Res Notes. 2009 Jun 1;2:95. doi: 10.1186/1756-0500-2-95 (PMC2702266; doi:10.1186/1756-0500-2-95)
Supplement: Additional file 2 — Table 2. Range, MIC50, MIC80 and MIC90 values (μg/ml) of L. tridentata extracts relative to clinical isolates and reference drug MIC90 values. [file 1756-0500-2-95-S2.doc]

Table 2. Range, MIC_50_, MIC_80_ and MIC_90_ values (μg/ml) of *L. tridentata* extracts relative to clinical isolates and reference drug MIC_90_ values.

|  | *L. tridentata* chloroformic extract | | | | *L. tridentata* methanolic extract | | | | Reference drugs | | | | | | | | | | | | |
| --- | --- | --- | --- | --- | --- | --- | --- | --- | --- | --- | --- | --- | --- | --- | --- | --- | --- | --- | --- | --- | --- |
| Organism | Range | MIC_50_ | MIC_80_ | MIC_90_ | Range | MIC_50_ | MIC_80_ | MIC_90_ | Cro | LV | Mer | Min | Pip/Taz | Pen | Va | Lz | Am | Amc | Ak | Cf | Caz |
| *A. baumannii* (n=25) | >250 | >250 | >250 | >250 | >250 | >250 | >250 | >250 | > 32 | > 8 | 16 | 2 | > 128 | NA | NA | NA | > 32 | > 32 | > 64 | > 32 | > 32 |
| *S. aureus* (n=25) | 12.5 - 100 | 125 | 125 | 125 | 12.5 - 250 | 125 | 125 | 250 | > 64 | > 32 | > 16 | ≤0.25 | > 16 | 8 | 2 | 2 | 16 | 8 | NA | NA | NA |
| *S. pneumoniae* (n=15) | 15.63 - 62.5 | 31.25 | 31.25 | 31.25 | 15.63 - 125 | 31.25 | 31.25 | 62.5 | 0.5 | 1 | 0.5 | 8 | 2 | 2 | 1 | 1 | 1 | 1 | NA | NA | NA |
| *E. faecalis* (n=15) | ≥250 | 250 | 250 | 250 | ≥250 | 250 | >250 | >250 | > 64 | 16 | 16 | > 8 | 4 | 4 | 4 | 2 | 2 | 1 | NA | NA | NA |

Ceftriaxone (Cro), Levofloxacin (LV), Meropenem (Mer), Minocycline (Min), Piperacillin Tazobactam (Pip/Taz), penicillin (Pen), Vancomycin (Va), Linezolid (Lz), Ampicillin (Am), Amoxicillin Clavulanic acid (Amc), Amikacin (Ak), Cefepime (Cf), Ceftazidime (Caz). NA: non assayed
